# P-92. Factors Associated with Outpatient IV Antibiotic Extension in Patients with Prosthetic Joint Infections

**DOI:** 10.1093/ofid/ofaf695.321

**Published:** 2026-01-11

**Authors:** Nicholas O Meade, Ryan P Mynatt, Ashley Logan, Takaaki Kobayashi, Nicole Leedy, Evelyn Villacorta Cari, Armaghan-E Rehman Mansoor

**Affiliations:** University of Kentucky, Lexington, KY; University of Kentucky, Lexington, KY; University of Kentucky HealthCare, Lexington, Kentucky; University of Kentucky, Lexington, KY; University of Kentucky, Lexington, KY; University of Kentucky, Lexington, KY; University of Kentucky, Lexington, KY

## Abstract

**Background:**

Guidelines recommend defined durations of antimicrobial therapy for prosthetic joint infections (PJI); however, therapy may be prolonged due to clinical or logistical factors. Extended antimicrobial use increases healthcare utilization, complicates care transitions, and exposes patients to unnecessary adverse event risk. We aimed to characterize the frequency, indications, and drivers of treatment extensions among patients receiving outpatient parenteral antimicrobial therapy (OPAT) for PJI.

**Table 1:**
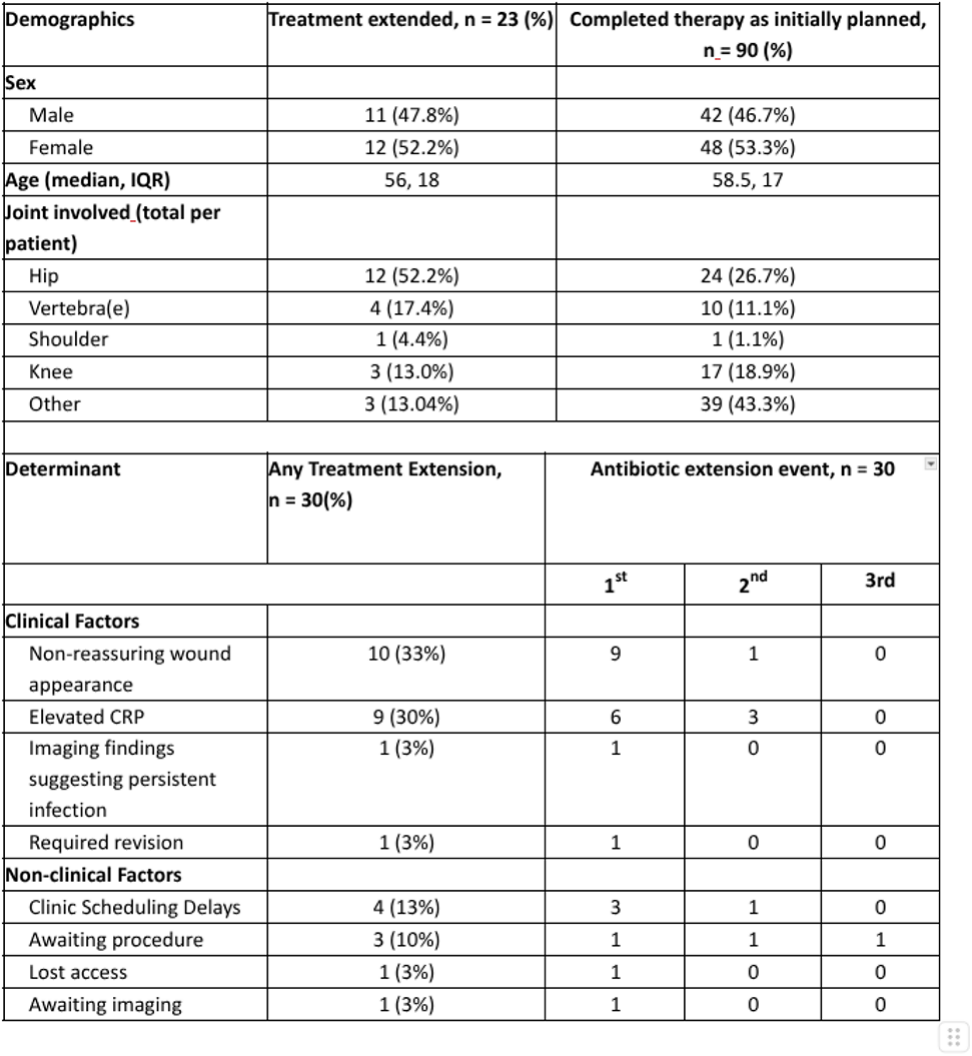
Demographics, and causes of treatment extensions in a cohort of patients receiving treatment for prosthetic joint infections via outpatient parenteral antimicrobial therapy (OPAT); CRP: C-reactive protein

**Methods:**

We conducted a retrospective cohort study of adults enrolled in an OPAT program between January 2023 and December 2024 for PJI. Demographic, clinical, and treatment data were abstracted from medical records. Treatment extension was defined as antimicrobial continuation beyond the planned duration. Reasons were categorized as clinical or non-clinical.

**Results:**

Among 113 patients, 90 (79.6%) completed therapy as planned, while 23 (20.4%) experienced extension, accounting for 30 events (median 1 per patient; range 1–3). Baseline characteristics were similar between groups: median age 58.5 years (IQR, 17) vs 58 years (IQR, 18) and male sex 47.8% vs 46.7% in the extension and non-extension groups, respectively. The most frequent clinical reasons were non-reassuring wound appearance (10/30, 33%) and persistently elevated C-reactive protein (CRP) (9/30, 30%). Non-clinical factors included clinic scheduling delays (4/30, 13%) and pending procedures (3/30, 10%). No extensions were attributed to antibiotic delivery, storage, or insurance issues.

**Conclusion:**

Approximately one in five patients receiving OPAT for PJI underwent treatment extension, most often due to wound findings or isolated inflammatory marker elevation without other infection evidence. Scheduling delays also contributed. These findings highlight stewardship opportunities, including streamlining outpatient scheduling and reconsidering inflammatory marker–driven extensions. Further research is needed to evaluate outcomes associated with extensions based solely on inflammatory markers, which may represent a target for reducing unnecessary antimicrobial exposure without compromising safety.

**Disclosures:**

All Authors: No reported disclosures

